# Intravenous Infusion of Autologous Mesenchymal Stem Cells Expanded in Auto Serum for Chronic Spinal Cord Injury Patients: A Case Series

**DOI:** 10.3390/jcm13206072

**Published:** 2024-10-11

**Authors:** Ryosuke Hirota, Masanori Sasaki, Satoshi Iyama, Kota Kurihara, Ryunosuke Fukushi, Hisashi Obara, Tsutomu Oshigiri, Tomonori Morita, Masahito Nakazaki, Takahiro Namioka, Ai Namioka, Rie Onodera, Yuko Kataoka-Sasaki, Shinichi Oka, Mitsuhiro Takemura, Ryo Ukai, Takahiro Yokoyama, Yuichi Sasaki, Tatsuro Yamashita, Masato Kobayashi, Yusuke Okuma, Reiko Kondo, Ryo Aichi, Satoko Ohmatsu, Noritaka Kawashima, Yoichi M. Ito, Masayoshi Kobune, Kohichi Takada, Sumio Ishiai, Toru Ogata, Atsushi Teramoto, Toshihiko Yamashita, Jeffery D. Kocsis, Osamu Honmou

**Affiliations:** 1 Department of Orthopaedic Surgery, School of Medicine, Sapporo Medical University, Sapporo 060-8556, Japan; ryosuke.hirota@yale.edu (R.H.); ryunosuke@sapmed.ac.jp (R.F.); osiben79@yahoo.co.jp (T.O.); morita.tomonori@sapmed.ac.jp (T.M.);; 2 Department of Neural Regenerative Medicine, Institute of Regenerative Medicine, School of Medicine, Sapporo Medical University, Sapporo 060-8556, Japan; nakazaki@sapmed.ac.jp (M.N.); m-takemura@kochi-u.ac.jp (M.T.); ryou@sapmed.ac.jp (R.U.); takahiro_yokoyama@sapmed.ac.jp (T.Y.);; 3 Department of Neurology, Yale University School of Medicine, New Haven, CT 06510, USA; 4 Department of Advanced Regenerative Therapeutics, School of Medicine, Sapporo Medical University, Sapporo 060-8556, Japan; 5 Department of Hematology, School of Medicine, Sapporo Medical University, Sapporo 060-8556, Japan; iyama@sapmed.ac.jp (S.I.);; 6 Department of Rehabilitation Medicine, School of Medicine, Sapporo Medical University, Sapporo 060-8556, Japan; 7 Section of Neuroregenerative Medicine and Rehabilitation, Hospital of National Rehabilitation Center for Persons with Disabilities, 4-1 Namiki, Tokorozawa 359-8555, Japan; 8 Biostatistics Division, Hokkaido University Hospital Clinical Research and Medical Innovation Center, N14W5, Kita-ku, Sapporo 060-8648, Japan; ito-ym@med.hokudai.ac.jp; 9 Department of Medical Oncology, Sapporo Medical University School of Medicine, Sapporo 060-8556, Japan; ktakada@sapmed.ac.jp; 10 Department of Rehabilitation Medicine, The University of Tokyo Hospital, 7-3-1, Hongo, Bunkyo-ku, Tokyo 113-8655, Japan; togata@g.ecc.u-tokyo.ac.jp; 11 Department of Neuroscience, Yale University School of Medicine, New Haven, CT 06510, USA

**Keywords:** mesenchymal stem cell, case series, chronic, spinal cord injury, clinical trials, intravenous

## Abstract

**Objective:** The safety, feasibility, and potential functional improvement following the intravenous infusion of mesenchymal stem cells (MSCs) were investigated in patients with chronic severe spinal cord injury (SCI). **Methods:** The intravenous infusion of autologous MSCs cultured in auto-serum under Good Manufacturing Practices (GMP) was administered to seven patients with chronic SCI (ranging from 1.3 years to 27 years after the onset of SCI). In addition to evaluating feasibility and safety, neurological function was evaluated using the American Spinal Injury Association Impairment Scale (AIS), International Standards for Neurological Classification of Spinal Cord Injury (ISCSCI-92), and Spinal Cord Independence Measure III (SCIM-III). **Results:** No serious adverse events occurred. Neither CNS tumors, abnormal cell growth, nor neurological deterioration occurred in any patients. While this initial case series was not blinded, significant functional improvements and increased quality of life (QOL) were observed at 90 and 180 days post-MSC infusion compared to pre-infusion status. One patient who had an AIS grade C improved to grade D within six months after MSC infusion. **Conclusions:** This case series suggests that the intravenous infusion of autologous MSCs is a safe and feasible therapeutic approach for chronic SCI patients. Furthermore, our data showed significant functional improvements and better QOL after MSC infusion in patients with chronic SCI. A blind large-scale study will be necessary to fully evaluate this possibility.

## 1. Introduction

Traumatic spinal cord injury (SCI) is a major cause of disability [1] and is being increasingly recognized as a global health concern [2]. The estimated number of individuals with traumatic SCI living in the United States is approximately 302,000, ranging from 255,000 to 383,000 [3]. In the chronic phase of SCI, functional recovery without therapeutic intervention is severely limited [4]. Therefore, new treatments are required to enhance residual function and improve the quality of life in patients with chronic SCI [5].

We previously reported functional improvements following cellular therapy with the intravenous infusion of bone marrow-derived mesenchymal stem cells (MSCs) in animal models of acute SCI. Even in the chronic phase of SCI, infused MSCs exert therapeutic efficacy, which is measured as an improvement in impaired locomotor function [6]. Potential therapeutic mechanisms for functional improvement in chronic SCI include the attenuation of blood–spinal cord barrier (BSCB) disruption [6], remyelination of demyelinated axons [6], enhancement of axonal projections in the injured spinal cord [6,7], and activation of the motor cortex [8].

The intravenous infusion of auto-serum-expanded autologous MSCs derived from bone marrows in patients has shown safety and potential therapeutic efficacy with acute SCI [9]. However, its clinical application in chronic SCI has not been explored.

Here, seven case reports of chronic SCI patients (ranging from 1.3 years to 27 years after onset of SCI with American Spinal Injury Association Impairment Scale (AIS) grades of C and D) are presented before and after the intravenous infusion of autologous bone marrow-derived MSCs. Safety and scoring on neurological scales were assessed, including the AIS grade, AIS motor score, AIS sensory score, and Spinal Cord Independence Measure (SCIM-III) score. Although a precise definition of the chronic phase of SCI has not been established, the operational definition is based on the stabilization of spontaneous recovery of function. This corresponds to approximately 6–12 months post-injury in humans; in rodent models, this translates to 1–2 months after injury [10,11].

## 2. Methods

### 2.1. Patients and Study Design

This study reports a case series of seven patients with chronic SCI as classified AIS grades C and D, who received autologous MSCs expanded in autologous serum. The patients included five men and two women aged 20–52 (mean 39.6 years). Seven chronic SCI patients were included in this study according to the following inclusion and exclusion criteria. The detailed inclusion and exclusion criteria are listed in the Supplementary Materials.

### 2.2. Preparation of Autologous Human Mesenchymal Stem Cells (MSCs): STR01

Autologous MSCs (STR01) derived from bone marrow were prepared using previously described protocols [9,12]. All cell culture procedures adhered to Good Manufacturing Practices (GMP) conditions, conducted by personnel with formal GMP training; the procedures were conducted in a facility where temperature, room air, pressure, and other environmental factors were strictly controlled. STR01 was made from each patient at a GMP cell-processing center and cryopreserved until use. The MSCs were cultured in autologous serum. Briefly, peripheral blood was collected multiple times to isolate serum from the patient. Blood cells were completely removed from the serum used for the culture. The bone marrow (30–60 mL) was collected from the posterior iliac crest of each patient under local anesthesia and was diluted with Dulbecco’s modified Eagle’s medium (Mediatech, Inc., Manassas, VA, USA or Cell Science & Technology Institute, Inc., Miyagi, Japan) supplemented with 10% autologous human serum, 2 mM L-glutamine (Sigma-Aldrich, UK or Cell Science & Technology Institute, Inc., Sendai, Japan), 100 U/mL penicillin–streptomycin (Sigma-Aldrich), was plated on 150 mm tissue culture dishes (AGC Techno Glass, Co., Ltd., Shizuoka, Japan) and incubated in a humidified atmosphere of 5% CO_2_ at 37 °C. When the cultures almost reached sub-confluence, adherent cells were detached with the trypsin–EDTA solution (Thermo Fisher Scientific, Waltham, MA, USA). The expanded autologous MSCs cultured in auto-serum were dissociated and diluted in 40 mL of storage solution [16 mL of RPMI (Thermo Fisher Scientific), 16 mL of auto-serum, 4 mL of low molecular dextran L (Otsuka Pharmaceutical Factory, Tokushima, Japan), 4 mL dimethyl sulphoxide (Nipro, Osaka, Japan)], frozen (Planer PLC, London, UK) and stored in a deep freezer (−150 °C) (Panasonic, Osaka, Japan) until use [9,12].

For the purpose of cell characterization and pathogen screening, the flow cytometry analysis of autologous MSCs was carried out as described in previous studies [9,12]. Briefly, cell suspensions were washed twice with phosphate-buffered saline. For direct assays, aliquots of MSCs at a concentration of 1 × 10^6^ cells/mL were immunolabeled at 4 °C for 30 min with the following antihuman antibodies: fluorescein isothiocyanate (FITC)-conjugated CD34 (Becton Dickinson, San Jose, CA, USA), R Phycoerythrin Cyanine 5.1 (PC5)-conjugated CD45 (Beckman Coulter, Brea, CA, USA), and phycoerythrin (PE)-conjugated CD105 (Becton Dickinson). Mouse immunoglobulin G1 (BioLegend, San Diego, CA, USA; Beckman Coulter) was utilized as an isotype-matched control. The labeled cells were analyzed using a flow cytometer (Cytomics FC500, Becton Coulter). The expanded cells underwent sterility testing to check for bacteria, mycoplasma, fungi, viruses (hepatitis B and C virus, adult T-cell leukemia virus, HIV, Parvovirus B19, Cytomegalovirus, EB virus), and endotoxin level assessments.

### 2.3. Study Procedures

All participants underwent an intensive rehabilitation (at least 80 min/weekday) protocol before MSC infusions to eliminate the potential effects of rehabilitation alone and to focus on the specific effects of MSCs. In brief, patients with chronic SCI who participated in this study received intensive rehabilitation (at least 80 min/week) for 4 weeks and continued intensive rehabilitation until they showed no further improvement in International Standards for Neurological Classification of Spinal Cord Injury (ISCSCI-92) scores [13] (>2 points) in the 2 weeks before MSC infusion. Therefore, we evaluated the therapeutic effects of MSC infusion in addition to those of rehabilitation therapy. If the patients required more than 2 weeks to reach a plateau in the ISCSCI-92 score, the intensive rehabilitation was extended until no further improvement was observed over the next two weeks.

On the day of infusion, cryopreserved units of MSCs (STR01) were thawed at the bedside using a 37 °C water bath, and were infused with saline to each patient over 30 min. The details regarding the concentrations and volumes of the injected cells are provided in the Supplemental Table. Each patient was closely observed during the autologous MSC infusions and for 24 h afterward. Monitoring included oxygen saturation, body temperature, electrocardiogram, blood pressure, pulse, and respiratory rate, both before and after the injection. Chest X-rays were also performed on each participant before and after cell infusion. At the 6-month mark, all participants underwent radiologic examinations and routine blood/urine tests to ensure there were no adverse effects.

### 2.4. Assessments

**Figure 1:**
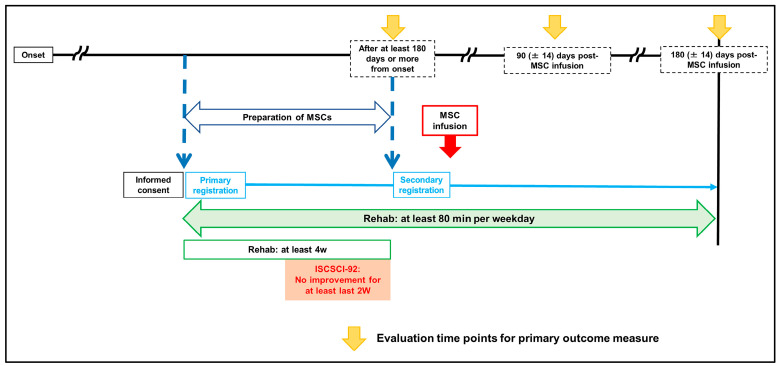
Clinical protocol.

Each participant’s status on SCI scales, including the AIS, ISCSCI-92, and Spinal Cord Independence Measure-III (SCIM-III) [14], was evaluated from 0 to 14 days before MSC infusion and at 90 (±14) and 180 (±14) days post-MSC infusion. The assessments were conducted by at least two board-certified orthopedic surgeons who were not blinded (Figure 1).

### 2.5. Statistical Analysis

Statistical analyses were conducted using JMP 11.1 for Windows (SAS Institute Inc., Cary, NC, USA). A one-way analysis of variance, followed by Bonferroni’s post hoc tests, was used to compare the differences in ISCSCI-92 and SCIM-III scores between the pre-infusion status and six months post-MSC infusion among the groups. The results are expressed as the mean  ±  standard error of the mean, with a *p*-value of less than 0.05 considered statistically significant.

### 2.6. Ethics

The study protocol was developed based on guidance from the Pharmaceuticals and Medical Devices Agency (PMDA) of Japan. This study was conducted in accordance with the Declaration of Helsinki and approved by the Institutional Review Boardof Sapporo Medical University (protocol code 29-17 and 9 November 2017). The trial was registered with the ID jRCT1090220329.

## 3. Results

### 3.1. Cell Preparation

Autologous human MSCs were expanded to 1.00–1.90 × 10^8^ cells within a relatively short culture period of 18.4 ± 2.5 days (Table 1). Cryopreservation allowed for detailed cell and pathogen characterization several days prior to cell infusion and resulted in higher cell viability (97.1 ± 0.3%) (Table 1). Sterility testing of MSCs showed no contamination, including bacteria, mycoplasma, fungi, hepatitis B and C virus, adult T-cell leukemia virus, HIV, Parvovirus B19, Cytomegalovirus, and EB virus, and endotoxin levels were non-pathogenic in all samples. Flow cytometric analysis confirmed that the MSCs exhibited CD34^−^, CD45^−^, and CD105^+^ cell surface phenotypes (Table 1).

### 3.2. Patient Characteristics

**Figure 2:**
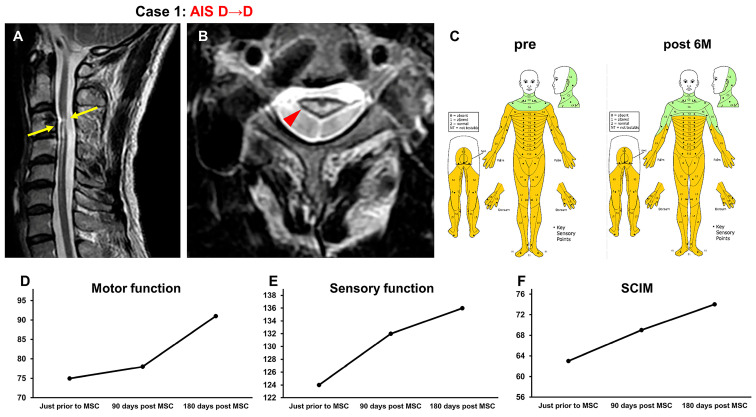
Case 1. T2-weighted MRI. (**A**) Sagittal arrows indicate the high-intensity areas. (**B**) Axial images. The arrowhead indicates the high-intensity areas. (**C**) Sensory function (pre, post 6M), (**D**) motor function, (**E**) sensory function, and (**F**) SCIM-III score.

**Figure 3:**
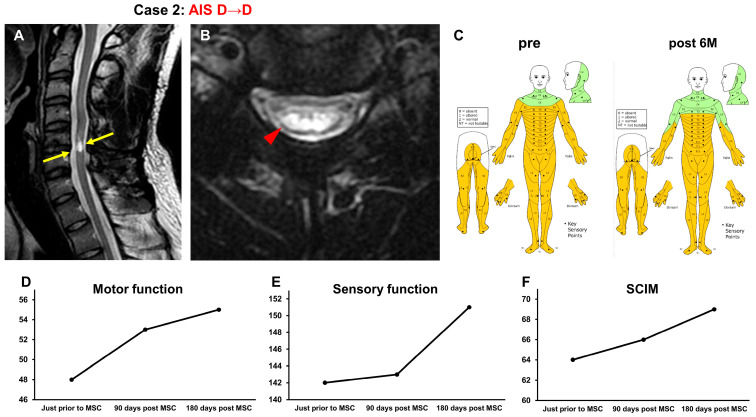
Case 2. T2-weighted MRI. (**A**) Sagittal arrows indicate the high-intensity areas. (**B**) Axial images. The arrowhead indicates the high-intensity areas. (**C**) Sensory function (pre, post 6M), (**D**) motor function, (**E**) sensory function, and (**F**) SCIM-III score.

**Figure 4:**
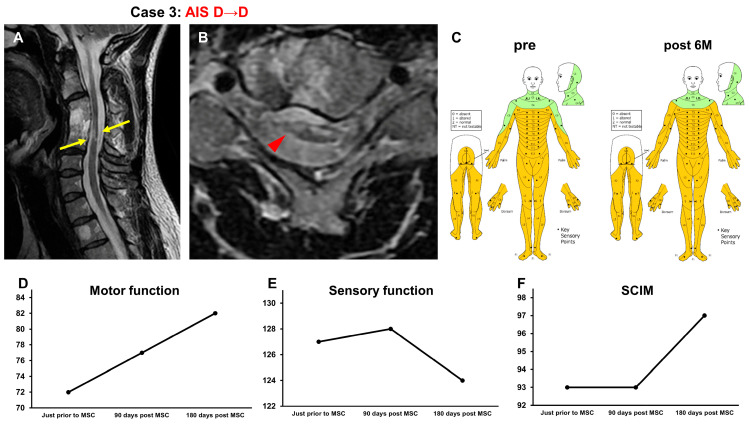
Case 3. T2-weighted MRI. (**A**) Sagittal arrows indicate the high-intensity areas. (**B**) Axial images. The arrowhead indicates the high-intensity areas. (**C**) Sensory function (pre, post 6M), (**D**) motor function, (**E**) sensory function, and (**F**) SCIM-III score.

**Figure 5:**
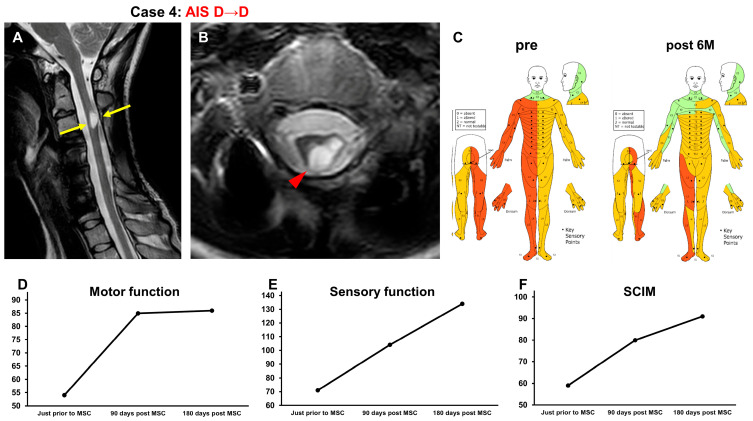
Case 4. T2-weighted MRI. (**A**) Sagittal arrows indicate the high-intensity areas. (**B**) Axial images. The arrowhead indicates the high-intensity areas. (**C**) Sensory function (pre, post 6M), (**D**) motor function, (**E**) sensory function, and (**F**) SCIM-III score.

**Figure 6:**
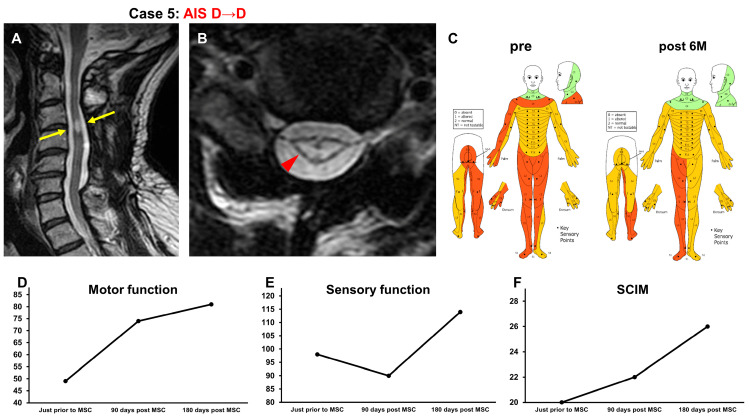
Case 5. T2-weighted MRI. (**A**) Sagittal arrows indicate the high-intensity areas. (**B**) Axial images. The arrowhead indicates the high-intensity areas. (**C**) Sensory function (pre, post 6M), (**D**) motor function, (**E**) sensory function, and (**F**) SCIM-III score.

**Figure 7:**
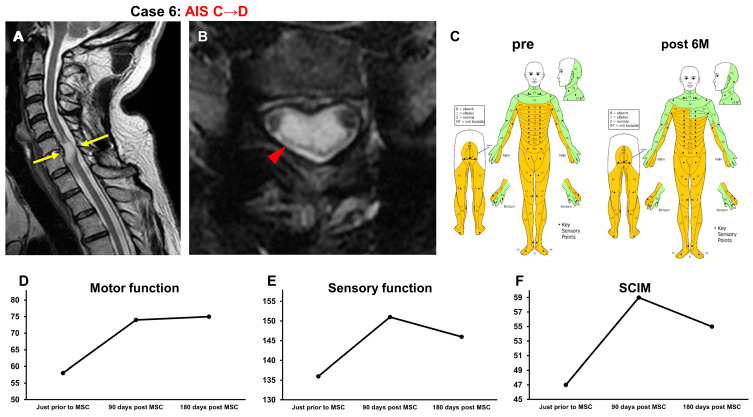
Case 6. T2-weighted MRI. (**A**) Sagittal arrows indicate the high-intensity areas. (**B**) Axial images. The arrowhead indicates the high-intensity areas. (**C**) Sensory function (pre, post 6M), (**D**) motor function, (**E**) sensory function, and (**F**) SCIM-III score.

**Figure 8:**
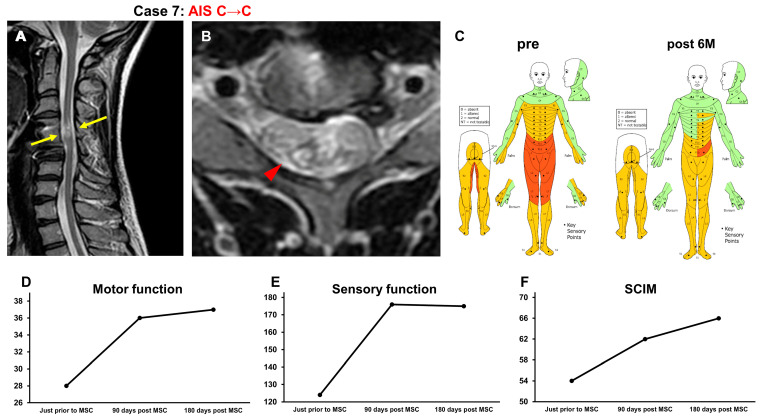
Case 7. T2-weighted MRI. (**A**) Sagittal arrows indicate the high-intensity areas. (**B**) Axial images. The arrowhead indicates the high-intensity areas. (**C**) Sensory function (pre, post 6M), (**D**) motor function, (**E**) sensory function, and (**F**) SCIM-III score.

The case histories of the seven patients are detailed in Table 2. This includes the case number, age, sex, level of injury, time from injury to MSC infusion (years), AIS grade immediately before MSC infusion, AIS grade 6 months after MSC infusion, changes in ISCSCI-92 (motor), changes in SCIM-III, and major adverse effects. For each patient, the individual AIS grade, ISCSCI-92 (motor and sensory scores), and SCIM-III scores over a 6-month period after MSC infusion are illustrated (Figure 2, Figure 3, Figure 4, Figure 5, Figure 6, Figure 7 and Figure 8).

### 3.3. Case Presentations

#### 3.3.1. Case 1 (Figure 2)

A 50-year-old man was injured when his head struck the ground during a backward somersault, resulting in neck hyperextension and cervical cord injury (NLI C3, AIS B). He underwent C3-4 posterior fixation 2 weeks after the SCI. Four years and four months after the onset of SCI, he received rehabilitation for 4 weeks. His recovery plateaued, and he was included in this study. At the time of transfer to Sapporo Medical University Hospital, T2-weighted MRI revealed a high-intensity area at C3–C4 (arrows in Figure 2A, arrowhead in Figure 2B) in the spinal cord (Figure 2A,B). His AIS grade was D, and his ISCSCI total motor score was 75. He received intensive rehabilitation for 4 weeks after primary registration and confirmed no further improvements in ISCSCI-92 scores for the last 2 weeks prior to MSC infusion at our hospital. He then received an intravenous infusion of 1.36 × 10^8^ autologous MSCs 5 years post SCI. Approximately 2 weeks following the MSC infusion, his trunk muscle strength improved, and he was able to sit up independently from a supine position. Another area that changed his function was improvement in his hand and finger dexterity. Improvements in upper limb function were also observed, enabling the patient to wash his hair, open the caps of plastic bottles, and use scissors to open envelopes. Spasticity had weakened, making it easier for him to walk. Six months after MSC infusion, the ISCSCI total motor score improved from 75 to 91 (Figure 2D), and the sensory score improved from 124 to 136 (Figure 2C,E). The SCIM-III score improved from 63 to 74 (Figure 2F).

#### 3.3.2. Case 2 (Figure 3)

A 20-year-old man was injured when he hit his head on the ground and hyperextended his neck while jumping into the ocean, which resulted in a C5 vertebral fracture and cervical cord injury (NLI C4, AIS B). He had severe sensory and motor disturbances on the right and left sides of the body, respectively. Two days after the injury, C4-6 posterior fusion was performed. One year and one month after the onset of SCI, he received intensive rehabilitation for 4 weeks. As his recovery reached a plateau, he was included in the study. At the time of his transfer to Sapporo Medical University Hospital, a T2-weighted MRI demonstrated spinal cord atrophy and high-intensity area in the spinal cord at C3–C5 (arrows in Figure 3A, arrowhead in Figure 3B) (Figure 3A,B). His AIS grade was D, and his ISCSCI total motor score was 48. He received intensive rehabilitation for 4 weeks and showed no further improvement in ISCSCI-92 scores for the last 2 weeks before MSC infusion in our hospital. He then received an intravenous infusion of 1.74 × 10^8^ autologous MSCs 1 year and 3 months post SCI. Approximately 1 month after MSC infusion, his trunk muscle strength improved, and he was able to raise his upper limbs in a sitting cross-legged posture and maintain a standing kneeling posture. Gradually, his ability to hold and move objects using his upper extremities improved. Six months after MSC infusion, the appearance of urinary and bowel movements enhanced incontinence. Additionally, the time required for defecation was reduced. The ISCSCI total motor score improved from 48 to 55 (Figure 3D), and the sensory score improved from 142 to 151 (Figure 3C,E). The SCIM-III score improved from 64 to 69 (Figure 3F).

#### 3.3.3. Case 3 (Figure 4)

A 48-year-old man sustained a C4 vertebral fracture and a cervical cord injury (NLI C4, AIS C) following a car accident. One week after the injury, C3-5 posterior fusion was performed. At 26 years and 8 months after the onset of the SCI, he underwent intensive rehabilitation for 4 weeks. As his recovery reached a plateau, he was included in this study. At the time of his transfer to Sapporo Medical University, T2-weighted MRI revealed spinal cord atrophy and a high-intensity area in the spinal cord at C4–C5 (arrows in Figure 4A, arrowhead in Figure 4B) in the spinal cord (Figure 4A,B). His AIS grade was D, and his ISCSCI total motor score was 69. He received intensive rehabilitation for 4 weeks and confirmed no further improvements in the ISCSCI-92 scores in the 2 weeks prior to MSC infusion in our hospital. He then received an intravenous infusion of 1.00 × 10^8^ autologous MSCs which was about 27 years after his SCI. Approximately 6 weeks post-MSC infusion, his left upper limb temperature, pain sensation disorder, and defecation function improved. Six months after MSC infusion, the patient was able to walk indoors without using a cane. His ISCSCI total motor score improved from 72 to 82 (Figure 4D), and his sensory score improved from 127 to 124 (Figure 4C,E). The SCIM-III score improved from 93 to 97 (Figure 4F).

#### 3.3.4. Case 4 (Figure 5)

A 28-year-old woman sustained a C5 vertebral fracture and cervical cord injury (NLI C 4, AIS A) in a car accident. No acute surgeries were performed. One year and ten months after the onset of the SCI, the patient underwent intensive rehabilitation for 5 weeks. Because her recovery reached a plateau, she was included in this study. At the time of transfer to Sapporo Medical University Hospital, T2-weighted MRI revealed a high-intensity area at the C3 level (arrows in Figure 5A, arrowhead in Figure 5B) in the spinal cord (Figure 5A,B). The AIS grade was D, and the ISCSCI total motor score was 54. She received intensive rehabilitation for 4 weeks and confirmed no further improvements in the ISCSCI-92 scores in the 2 weeks prior to MSC infusion in our hospital. She then received an intravenous infusion of 1.22 × 10^8^ autologous MSCs 2 years and 1 month after SCI. One day after MSC infusion, the sensory disturbances in the right hemisphere improved. In addition, approximately 1 week after MSC administration, muscle strength on the left side, where motor paralysis was severe, started to improve. Six months after the intravenous infusion of MSCs, she could stand independently and walk long distances using a cane. She was able to safely climb stairs using a handrail. She was able to dress herself without assistance. Her ISCSCI total motor score improved from 54 to 86 (Figure 5D), and her sensory score improved from 71 to 134 (Figure 5C,E). The SCIM-III score improved from 59 to 91 (Figure 5F).

#### 3.3.5. Case 5 (Figure 6)

A 49-year-old man suffered a C3 dislocation fracture/cervical cord injury (NLI C3; AIS B) in a motorcycle accident. The patient underwent posterior fusion 2 weeks after the injury. Two years and eight months after the onset of SCI, he received intensive rehabilitation for 6 weeks. The patient’s recovery reached a plateau, and he was included in this study. At the time of transfer to Sapporo Medical University, T2-weighted MRI revealed a high-intensity area at C3–C4 (arrows in Figure 6A, arrowhead in Figure 6B) in the spinal cord (Figure 6A,B). His AIS grade was D, and his ISCSCI total motor score was 49. He received intensive rehabilitation for 4 weeks and confirmed no further improvements in the ISCSCI-92 scores in the 2 weeks prior to MSC infusion in our hospital. He then received an intravenous infusion of 1.88 × 10^8^ autologous MSCs 2 years and 9 months after SCI. Approximately 1 week after MSC infusion, improvements in the motor function of the shoulder and elbow and fine motor function of the fingers were observed. Consequently, he started eating on his own using an assistive device. Six months after treatment, the patient could wash his face and brush his teeth by himself using a self-help device and was able to turn over in bed. The patient was then transferred to a wheelchair under supervision. The ISCSCI total motor score improved from 49 to 81 (Figure 6D), and the sensory score improved from 98 to 114 (Figure 6C,E). The SCIM-III score improved from 20 to 26 (Figure 6F).

#### 3.3.6. Case 6 (Figure 7)

A 52-year-old man sustained a C6 dislocation fracture and cervical cord injury (NLI C5, AIS A). The patient underwent C5-Th1 posterior fusion on the same day as the injury. Three years and nine months after the onset of SCI, he received intensive rehabilitation for 6 weeks. The patient’s recovery reached a plateau and was included in this study. At the time of transfer to Sapporo Medical University, T2-weighted MRI revealed a high-intensity area at C6–C7 (arrows in Figure 7A, arrowhead in Figure 7B) in the spinal cord (Figure 7A,B). His AIS grade was C, and his ISCSCI total motor score was 58. He received intensive rehabilitation for 4 weeks and confirmed no further improvements in the ISCSCI-92 scores in the 2 weeks prior to MSC infusion in our hospital. He then received an intravenous infusion of 1.90 × 10^8^ autologous MSCs 4 years post SCI. Approximately 1 month following the MSC infusion, hand and lower limb motor function improvements were observed. In addition, the voluntary contraction of the anus, which had not been previously confirmed, was honored at the 90-day post-administration evaluation. Six months after the MSC infusion, the amount of assistance required for bathing decreased, and the patient was able to transfer from bed to wheelchair independently. The AIS score improved from C to D, the ISCSCI total motor score improved from 58 to 75 (Figure 7D), and the sensory score improved from 136 to 146 (Figure 7C,E). The SCIM-III score improved from 47 to 55 (Figure 7F).

#### 3.3.7. Case 7 (Figure 8)

A 30-year-old woman sustained a C4 dislocation fracture and cervical cord injury (NLI C4, AIS B). C4-5 anterior fusion was performed the day after the injury. At 18 years and 9 months after the onset of SCI, the patient underwent intensive rehabilitation for 7 weeks. The patient’s recovery reached a plateau, and she was included in this study. At the time of her transfer to Sapporo Medical University, T2-weighted MRI revealed spinal cord atrophy and a high-intensity area in the spinal cord at C4–C5 (arrows in Figure 8A, arrowhead in Figure 8B) in the spinal cord (Figure 8A,B). The AIS grade was C, and the ISCSCI total motor score was 28. She received intensive rehabilitation for 4 weeks and confirmed no further improvements in the ISCSCI-92 scores in the 2 weeks prior to MSC infusion in our hospital. She received an intravenous infusion of 1.30 × 10^8^ autologous MSCs 18 years after SCI. Approximately one month post MSC infusion, improvement in the fine motor function of the fingers and acquisition of trunk stability were observed. Consequently, the patient was able to drive a wheelchair outdoors. Previously, she needed assistance in bathing but became independent by adjusting her environment. In addition, she was able to cook. Six months after the MSC infusion, her ISCSCI total motor score improved from 28 to 37 (Figure 8D), and her sensory score improved from 124 to 175 (Figure 8C,E). The SCIM-III score improved from 54 to 66 (Figure 8F).

### 3.4. Clinical Data

Seven patients (Table 2) aged 20 to 52 years (average: 39.6 ± 5.0, median: 48) of both genders were included in this study. The intravenous infusion of autologous MSCs were performed 1.3–27 years post-SCI. No serious adverse events occurred following MSC injection. Adverse events related to the protocol included anemia following peripheral blood collection in six cases and localized pain at the bone marrow aspiration site in one case immediately after the procedure. All protocol-related adverse events occurred prior to MSC infusion, and thus, no MSC-related adverse events were observed in the current study.

We conducted the final assessment approximately 180 days after MSC infusion. One of the two patients initially classified as AIS C before MSC treatment improved to AIS D 6 months post-infusion. None of the participants with AIS D (n = 5) changed their AIS grade at 6 months after MSC infusion (Table 3).

**Figure 9:**
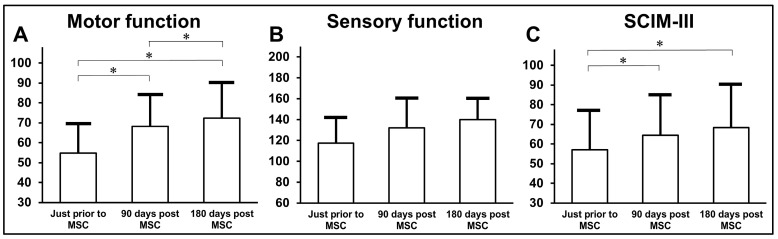
Outcome measure scores according to AIS C classification ((**A**): motor; (**B**): sensory; (**C**): SCIM-III) prior to MSC infusion, 90 and 180 days post-MSC infusion.

The patients showed gradual increases in motor scores, with statistically significant improvements observed at 90 and 180 days post-MSC infusion compared with those before MSC infusions (Figure 9A). Additionally, the motor score at 180 days post-MSC infusion was higher than that at 90 days post-MSC infusion. Sensory scores were evaluated before MSC infusion and at 90 and 180 days post-MSC infusion (Figure 9B). The patients displayed gradual increases in SCIM-III scores, with statistical significance at 90 and 180 days post-MSC infusion compared to pre-MSC infusions (Figure 9C).

**Figure 10:**
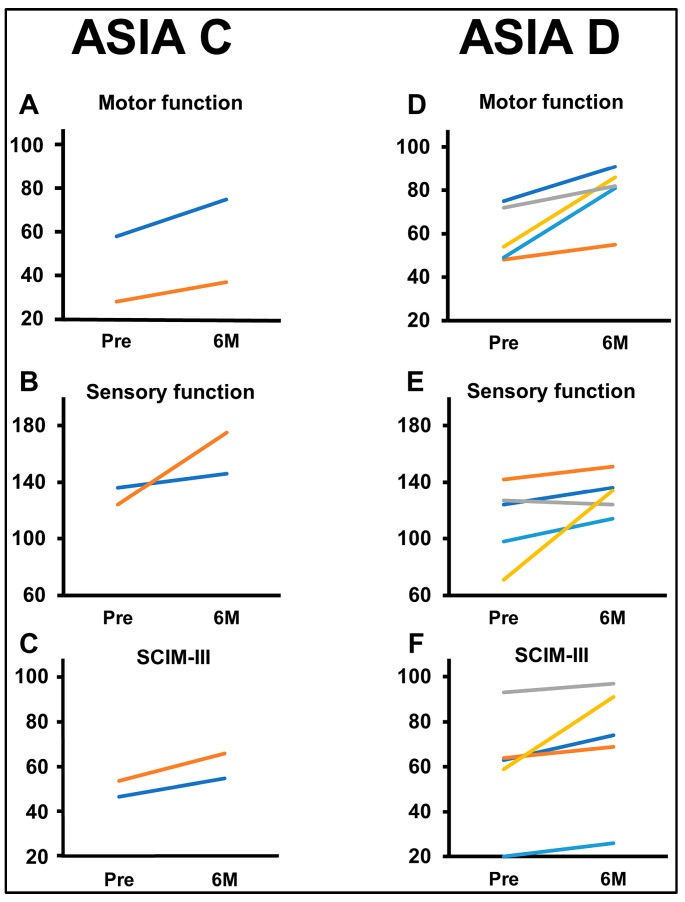
Comparison of outcome measure scores before infusion and six months post-MSC infusion based on AIS classification ((**A**,**D**): motor; (**B**,**E**): sensory; (**C**,**F**): SCIM-III).

Individual changes are presented in the ISCSCI-92 scores, including motor and sensory scores and SCIM-III scores, in patients with AIS C and AIS D prior to MSC infusion and 180 days after MSC infusion (Figure 10). Motor (Figure 10A) and sensory (Figure 10B) functions at six months (±14 days) showed improvements in all patients compared to their pre-MSC infusion scores. In AIS D, the motor scores (Figure 10D) at six months (±14 days) were higher than the scores prior to MSC infusion in all patients. Sensory scores (Figure 10E) at six months (±14 days) were higher than the scores prior to MSC infusion in four patients. SCIM-III total scores at six months (±14 days) were higher than pre-infusion scores in all patients across both groups (Figure 10C: AIS C; Figure 10F: AIS D).

## 4. Discussion

In this case series, the results are presented and show that infused autologous bone marrow-derived MSCs cultured in auto-serum into seven patients with chronic SCI (AIS C and D) is safe, feasible, and potentially therapeutically efficacious. None of the patients had MSC-related AE, CNS tumors, abnormal cell growth, or neurological deterioration. In terms of feasibility, the practicality of our protocol, including the recruitment of patients with chronic SCI, study management, regulatory compliance, and ethical assurance, was affirmed. Additionally, the expansion of autologous human MSCs in autologous human serum and cryopreservation before injection into patients was shown to be practical [9].

The average scores for motor functions at 3 and 6 months after MSC infusion showed statistically significant improvements compared to pre-MSC infusion. The statistically significant increase in SCIM-III scores indicates the potential enhancement of quality of life (QOL) [15] following the intravenous injection of MSCs. Together with detailed case presentations, these observations highlight the potential therapeutic efficacy and improvements in QOL after MSC infusion in the current study. It should be noted that no worsening of neuropathic pain [16] occurred at this injury level or below.

Our rationale for using MSCs in this study is based on extensive preclinical research involving animal models of neurological diseases, including chronic SCI. In our research, rats were studied with contusive SCI using a “chronic” SCI model after 10 weeks [6]. The MSC-treated group observed significantly greater motor recovery than the vehicle-treated group. Several potential therapeutic mechanisms could underlie MSC infusion in chronic SCI. One possible mechanism is restoring the disrupted blood–spinal cord barrier (BSCB) using infused MSCs. Because it is important to reduce the breakdown of the BSCB after SCI. Disrupted BSCB leads to secondary tissue damage, including immune cell infiltration and the release of toxic molecules, resulting in neuronal loss, axon damage, and demyelination [17,18]. This BSCB compromise is known to persist for an extended time after SCI [19,20]. The spectrophotometric quantification of Evans blue dye leakage into the parenchyma demonstrated a significant reduction in BSCB disruption in the MSC group compared to the vehicle group, in agreement with previous studies [21,22]. Additionally, immunohistochemical analyses using RECA-1 and PDGFR-β antibodies displayed increased microvasculature and repair-neovascularization in MSC-infused rats [6]. Secondly, the remyelination of demyelinated axons could be another mechanism [23]. The MSC group exhibited extensive remyelination around the lesion center and increased the sprouting of corticospinal tracts and serotonergic fibers compared to the vehicle-infused group [6]. Thirdly, infused MSCs may improve axonal connections in the injured spinal cord [6] and be the possibility of bypassing neural post-SCI gaps [24]. The enhancement of axonal projections from the dorsal corticospinal tract to axons in the lateral funiculus on both sides of the lesion core suggests that fine caliber pre-existing axons that are not readily observed might increase in diameter and become detectable [7]. This was demonstrated by tracing descending motor axons with an adeno-associated vector [25] combined with advanced tissue-clearing techniques [26]. These enlarged axons may contribute to improved conduction to motor neurons below the SCI site following intravenous infusion of MSCs. Fourthly, infused MSCs may activate certain brain regions [8]. There is an enhancement of remote gene expression responses in the brain following intravenous infusion of MSCs in rat SCI, suggesting that infused MSCs might turn on the activation in the remote area, i.e., the motor cortex in the SCI. Fifteen coding genes were identified in the motor cortices after SCI between the MSC- and vehicle-infused SCI animals, indicating that the overall gene signature in these remote neural tissues may be triggered to initiate additional downstream gene expression in order to restore neuronal function, promoting axonal outgrowth and synaptic connectivity, following the intravenous infusion of MSCs. Taken together, these results indicate that MSC infusion causes functional improvement associated with structural changes in the chronically injured spinal cord via multiple orchestrated processes by the accumulated MSCs, including the stabilization of the BSCB, axonal sprouting/regeneration, remyelination, remote effects, and the facilitation of neural connections, which lead to the enhancement of neural plasticity and uncovering latent neural pathways.

The intravenous infusion of MSCs might facilitate neural plasticity not only in the spinal cord [7] but also in the CNS [12]. Several experimental studies in the brain have provided corroborative evidence supporting the role of infused MSCs in enhancing neural plasticity under pathological conditions. Infused MSCs accumulate at the site of injury lesions [6], facilitate neuronal connections between ischemic and non-ischemic brains, and promote global neural plasticity. In the developing brain, the intravenous MSC infusion facilitates functional improvements and promotes brain tissue growth in the unaffected brain hemispheres. Several supporting pieces of evidence have shown improved neural plasticity following the intravenous infusion of MSCs in various neural disorder models [9,12].

While this initial case series was unblinded and uncontrolled, patients with chronic SCI showed improved neurological function following MSC infusion, demonstrating its safety and feasibility. This case series represents an early study with a small number of patients with chronic SCI. This study has several limitations, including being unblinded and uncontrolled [27]. Observer bias, the potential contribution of surgical intervention to recovery in cases where it occurred, and the possibility of spontaneous recovery cannot be ruled out.

## 5. Conclusions

In this study, detailed clinical records are reported for seven patients with chronic SCI before and after the infusion of autologous MSCs expanded in auto-serum. The reported observations suggest the safety and feasibility of MSC infusion and provide initial data suggesting potential functional improvements. This study highlights the importance of future large-scale, placebo-controlled clinical studies to determine the efficacy of MSC infusion in patients with chronic SCI.

## Figures and Tables

**Table 1:** Characteristics of processed MSCs.

Case Number	Total Cell Numbers (Cells)	Concentration of Injected Cells (Cells/mL)	Volume (mL)	CD105 (%)	CD34 (%)	CD45 (%)	Cell Viability (%)	Number of Culture Days (Day)
1	1.36 × 10^8^	3.40 × 10^6^	40	100.0	0.2	0.7	94.7	15
				99.9	0.1	0.7	97.8	
2	1.74 × 10^8^	4.35 × 10^6^	40	99.7	0.0	0.2	95.7	15
				99.9	0.0	0.0	96.8	
3	1.00 × 10^8^	2.50 × 10^6^	40	99.6	0.1	0.0	96.4	22
				99.8	0.0	0.2	97.1	
4	1.22 × 10^8^	3.05 × 10^6^	40	100.0	0.0	1.2	97.9	15
				99.9	0.0	1.4	96.7	
5	1.88 × 10^8^	4.70 × 10^6^	40	100.0	0.0	1.2	97.9	15
				100.0	0.3	0.9	98.5	
6	1.90 × 10^8^	4.75 × 10^6^	40	99.9	0.1	0.4	98.4	15
				100.0	0.1	0.4	97.7	
7	1.30 × 10^8^	3.25 × 10^6^	40	98.0	0.0	0.0	97.0	32
				98.0	0.0	0.0	97.0	

**Table 2:** Summary of cases.

Case Number	Age (y.o)	Sex	Level of Injury	Time of Injury until MSC Infusion	AIS Grade just before MSC Infusion	AIS Grade 6 Months Post-MSC Infusion	Difference in Motor Function	Difference in SCIM-III	Major Adverse Effects
1	50	male	C4	4.7 y	D	D	16	11	None
2	20	male	C4	1.3 y	D	D	7	5	None
3	48	male	C5	27 y	D	D	10	4	None
4	28	female	C3	2.1 y	D	D	32	32	None
5	49	male	C4	2.9 y	D	D	32	6	None
6	52	male	C7	4.0 y	C	D	17	8	None
7	30	female	C6	18.0 y	C	C	9	12	None

**Table 3:** AIS impairment class between before infusion and after 6 months.

	AIS at 180 Days Post-MSC Infusion		
AIS prior to MSC infusion		AIS C	AIS D
	AIS C	50% (1/2)	50% (1/2)
	AIS D	0%	100% (5/5)

## Data Availability

De-identified individual participant data and relevant supporting clinical study documents are available upon formal request from qualified scientific and medical researchers.

## Supplementary Materials

The following supporting information can be downloaded at: https://www.mdpi.com/article/10.3390/jcm13206072/s1, list of detailed inclusion and exclusion criteria.

## Abbreviations

AE	Adverse events
AIS	American Spinal Injury Association Impairment Scale
BSCB	Blood–spinal cord barrier
ISCSCI	International Standards for Neurological Classification of Spinal Cord Injury
MSC	Mesenchymal stem cells
PMDA	Pharmaceuticals and Medical Devices Agency
QOL	Quality of life
SCI	Spinal cord injury
SCIM	Spinal Cord Independence Measure
