# Supplementary material for: Intravenous Infusion of Autologous Mesenchymal Stem Cells Expanded in Auto Serum for Chronic Spinal Cord Injury Patients: A Case Series
Source: J Clin Med. 2024 Oct 11;13(20):6072. doi: 10.3390/jcm13206072 (PMC11509003; doi:10.3390/jcm13206072)
Supplement: Supplementary file 1 [file jcm-13-06072-s001.zip › JCM_STR01-04_Supplementary Table.pdf]

Supplementary Table

No.	Total Cell Numbers (cells)	Concentration of Injected Cells (cells/mL)	Volume (mL)	CD105 (%)	CD34 (%)	CD45 (%)	Cell viability (%)	Number of culture days (day)
SCI02-02	1.36×10^8	3.40×10^6	40	100.0	0.2	0.7	94.7	15
				99.9	0.1	0.7	97.8	
SCI02-06	1.74×10^8	4.35×10^6	40	99.7	0.0	0.2	95.7	15
				99.9	0.0	0.0	96.8	
SCI02-08	1.00×10^8	2.50×10^6	40	99.6	0.1	0.0	96.4	22
				99.8	0.0	0.2	97.1	
SCI02-11	1.22×10^8	3.05×10^6	40	100.0	0.0	1.2	97.9	15
				99.9	0.0	1.4	96.7	
SCI02-13	1.88×10^8	4.70×10^6	40	100.0	0.0	1.2	97.9	15
				100.0	0.3	0.9	98.5	
SCI02-14	1.90×10^8	4.75×10^6	40	99.9	0.1	0.4	98.4	15
				100.0	0.1	0.4	97.7	
SCI02-16	1.30×10^8	3.25×10^6	40	98.0	0.0	0.0	97.0	32
				98.0	0.0	0.0	97.0	
