# Supplementary material for: Intravenous Infusion of Autologous Mesenchymal Stem Cells Expanded in Auto Serum for Chronic Spinal Cord Injury Patients: A Case Series
Source: J Clin Med. 2024 Oct 11;13(20):6072. doi: 10.3390/jcm13206072 (PMC11509003; doi:10.3390/jcm13206072)
Supplement: Supplementary file 1 [file jcm-13-06072-s001.zip › JCM_STR01-04_Supplementary_Appendix.pdf]

Supplementary Appendix:

[Inclusion Criteria at the Time of First Registration]

(1) Spinal cord injury, where partial damage to the spinal cord is recognized by imaging diagnosis (incomplete separation).

(2) Classified as A-D of the ASIA impairment scale, the motor function score of ISCSCI-92 is 90 points or less in the case of cervical cord injury or 98 points or less in the case of thoracic and lumbar cord injury

(3) Age ≧ 18 years

(4) Rehabilitation can be performed in > at least 80 minutes per weekday.

(5) Written informed consent was obtained from the participants. If a participant was unable to write, written informed consent was obtained from the legal representative.

[Inclusion Criteria at the Time of Second Registration]

(1)180 days or more after onset of spinal cord injury

(2) After rehabilitating more than 80 minutes per weekday as much as possible over the past one month or more, there has been no improvement of ISCSCI-92 over the past 2 weeks; * Definition of improvement of ISCSCI-92: improvement of 2 points or more as total points of ISCSCI-92. When the sensory function was likely to change, we used the average of the values evaluated consecutively for 3 days.

(3) Classified as A-D on the ASIA impairment scale

(4) Patients who were ready for the infusion of STR-01 that satisfied the acceptance criteria.

[Exclusion criteria at the time of first registration]

(1) Severe consciousness disturbances (Japan Coma Scale 200 and 300)

(2) Severe respiratory disorder

(3) Diagnosis of hepatitis B, hepatitis C, and syphilis through initial screening

(4) Pancytopenia (a white blood cell concentration of <2000 cells/μL, hemoglobin concentration of <10.0 g/dl, or platelet concentration of <100,000 platelets/μL)

(5) History of neoplasms (except complete response), severe diseases of the blood and blood-forming organs, certain disorders involving the immune mechanism, severe mental and behavioral disorders, severe diseases of the nervous system, severe congenital malformations, deformations, and chromosomal abnormalities.

(6) Past history of penicillin and streptomycin allergies or other severe allergies (shock or anaphylactic symptoms).

(7) Poor general condition due to endocrine, nutritional, and metabolic diseases; uncontrollable mental disorders; diseases of the nervous system (refractory epilepsy); diseases of the circulatory system (uncontrollable and refractory heart failure, moderate or severe valvular heart disorder, uncontrollable and refractory atrial fibrillation, refractory atrial and ventricular thrombi, a history of ischemic heart disease with percutaneous coronary intervention within the past 12 months, and serious arrhythmia); diseases of the respiratory system; diseases of the digestive system; diseases of the musculoskeletal system and connective tissue; diseases of the genitourinary system (dialysis); injury; poisoning; and certain other consequences of external causes

(8) Severe poly-injury or multiple organ failure

(9) Severe pre-existing spinal cord or spinal column disease (severe osteoporosis, spinal cord tumor, spinal cord vascular malformation, and severe syringomyelia)

(10) Moyamoya disease, cerebral aneurysms, and other vascular malformations have a high risk of rupture or cerebral embolism, including a history of these issues.

(11) 70% or more of arterial stenosis or dissection causes cerebral infarction even after revascularization (except complete thrombotic occlusion).

(12) Severe arteriosclerotic changes and calcification of the blood vessels of the head and neck.

(13) Possible preoperative uncontrollable hypertension under depressor therapy (systolic

pressure > 140 mmHg, diastolic pressure > 90 mmHg)

(14) Participation in another clinical trial related to any organ or a history of cell therapy

(15) Pregnant or possibly pregnant, nursing women, those who plan to become pregnant during the study period, or male patients who wish their partner to get pregnant

(16) Other patients judged by investigators holding a medical license were inappropriate for the study.

[Exclusion criteria at the time of the second registration]

(1) Severe consciousness disturbances (Japan Coma Scale 200 and 300)

(2) Severe respiratory disorder

(3) Diagnosis of hepatitis B and C, HIV, human T-lymphotropic virus 1, syphilis, or human parvovirus B19 infection via detailed examination

(4) Neoplasms (except complete response), severe diseases of the blood and blood-forming organs, certain disorders involving immune mechanisms, severe mental and behavioral disorders, severe diseases of the nervous system, severe congenital malformations, deformations, and chromosomal abnormalities.

(5) Penicillin and streptomycin allergies and other severe allergies (shock and anaphylactoid symptoms)

(6) Poor general condition due to endocrine, nutritional, and metabolic diseases; uncontrollable mental disorders; diseases of the nervous system (refractory epilepsy), diseases of the circulatory system (uncontrollable and refractory heart failure, moderate or severe valvular heart disorder, uncontrollable and refractory atrial fibrillation, refractory atrial and ventricular thrombi, a history of ischemic heart disease and percutaneous coronary intervention within the past 12 months, and serious arrhythmia); diseases of the respiratory system; diseases of the digestive system; diseases of the musculoskeletal system and connective tissue; diseases of the genitourinary system (dialysis); injury; poisoning; and certain other consequences of external causes

(7) Severe poly-injury or multiple-organ failure

(8) Severe spinal cord or spinal column disease (severe osteoporosis, spinal cord tumor, spinal cord vascular malformation, and severe syringomyelia)

(9) Moyamoya disease, cerebral aneurysms, and other vascular malformations have a high risk of rupture or cerebral embolism, including a history of these issues.

(10) More than 70% stenosis of the main cerebral arteries and cervical carotid and vertebral arteries, even after revascularization (except for complete occlusion and a healed dissecting artery) or arterial dissection

(11) Severe arteriosclerotic changes and calcifications

(12) Possible preoperative uncontrollable hypertension under depressor therapy (systolic pressure > 140 mmHg, diastolic pressure > 90 mmHg)

(13) Participation in another clinical trial related to any organ or a history of cell therapy

(14) Pregnant or possibly pregnant nursing women, those planning to become pregnant during the study period, or male patients wishing to become pregnant

(15) Other patients judged by investigators holding a medical license were inappropriate for the study.
